# MR Imaging of Migrated Nucleus Pulposus after Collagenase Injection of Lumbar Disc Herniation: A Case Report

**DOI:** 10.5334/jbsr.2777

**Published:** 2022-08-11

**Authors:** Bai Jin Tao Sun, Bing Li, Xu Feng, Han Feng Yang

**Affiliations:** 1 Affiliated Hospital of North Sichuan Medical College, CN

**Keywords:** lumbar disc herniation (LDH), collagenase, nucleus pulposus prolapsus, MR imaging (MRI)

## Abstract

Magnetic resonance images (MRI) of migrated nucleus pulposus after collagenase treatment of lumbar disc herniation are rarely published. Here, we describe a 65-year-old woman with L5-S1 intervertebral disc herniation on the rear left. The patient was treated with a lumbar disc collagenase injection, and the pain was relieved. Two weeks later, the patient suddenly developed pain again after engaging in weight-bearing activity. Lumbar MRI showed a nodule in the spinal canal at the L5-S1 level. The patient underwent surgical treatment two days later. Pathology showed that the nodule was nucleus pulposus tissue.

**Teaching point:** It is important to understanding the MR manifestations of migrated nucleus pulposus after collagenase treatment to prevent such misdiagnosis.

## Introduction

Lumbar disc herniation (LDH) is defined as a localized displacement of disc tissues beyond the limits of the intervertebral disc space and can be classified as protrusions or extrusions; the latter include sequestration, migration, and intravertebral herniations, based on the shape of the displaced tissues [1]. MRI is a reliable method of confirming suspected LDH. Collagenase injection is one of the more common minimally invasive methods used to treat LDH [2]. It has been proved that collagenase can relieve compression of the dural sac and irritation of nerve roots [3]. However, specific complications may occur after collagenase injection, such as pain, fever, cauda equina syndrome, disc herniation, anaphylaxis, bleeding, and neurologic complications [4]. Migration of the nucleus pulposus after collagenase injection to correct lumbar disc herniation has a low incidence, and its MR manifestations can be misdiagnosed. Here, we report one case to facilitate understanding of the MR manifestations of migrated nucleus pulposus after collagenase treatment to prevent such misdiagnosis.

## Case Report

### History and Presentation

**Figure 1:**
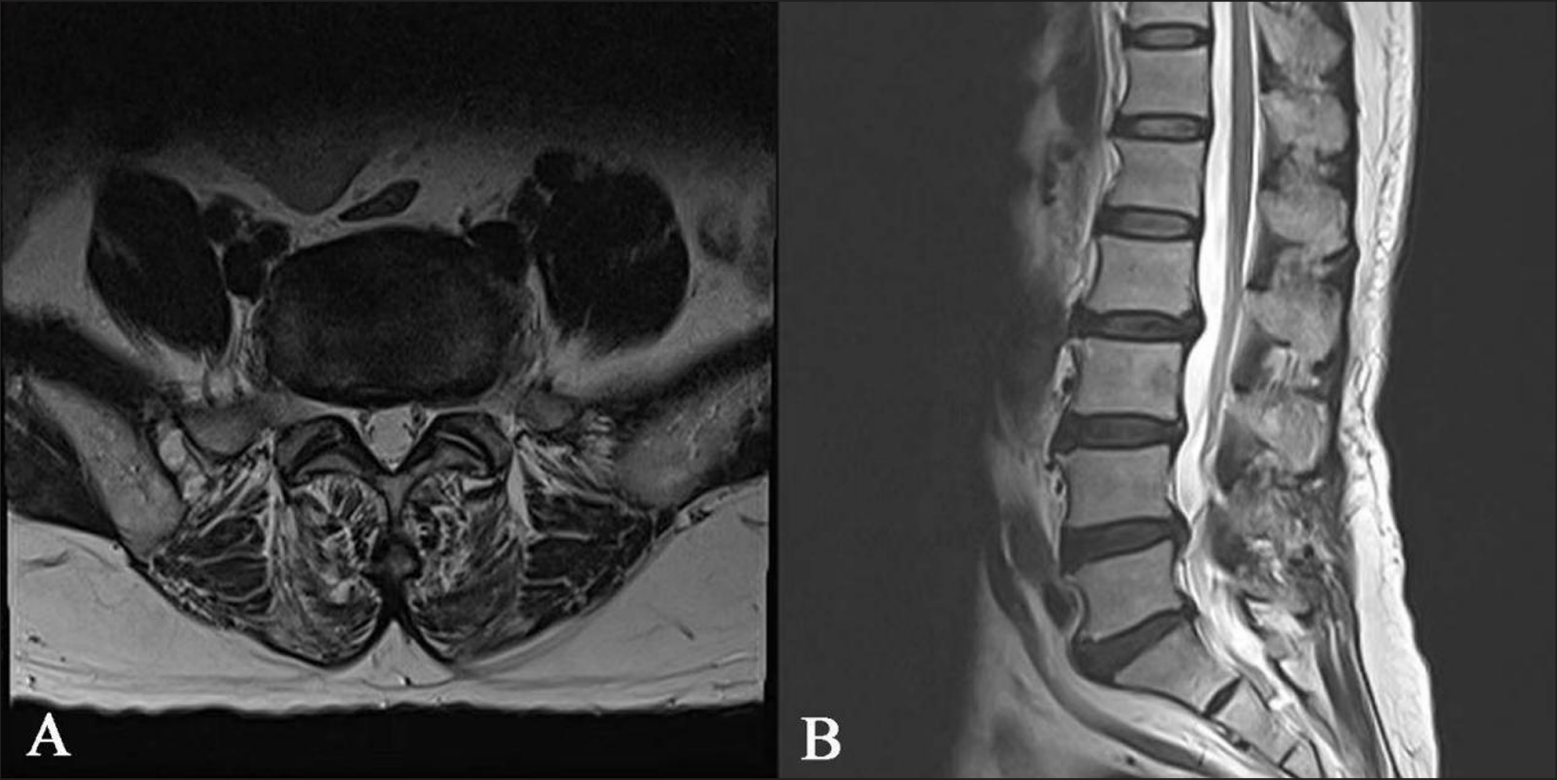
Preoperative: T2-weighted axial **(A)** and sagittal **(B)** MR images without contrast.

A 65-year-old woman presented with mild left lower extremity pain for 4–5 weeks that worsened after about one week. The visual analog scale (VAS) pain score was 7. No sensory disturbances and upper motor neuron signs were documented. A lumbar spine MR scan showed that the L5–S1 intervertebral disc was slightly herniated to the rear left (Figure 1). The patient underwent a lumbar L5–S1 intervertebral disc collagenase injection 28 hours after presentation.

### Operation

**Figure 2:**
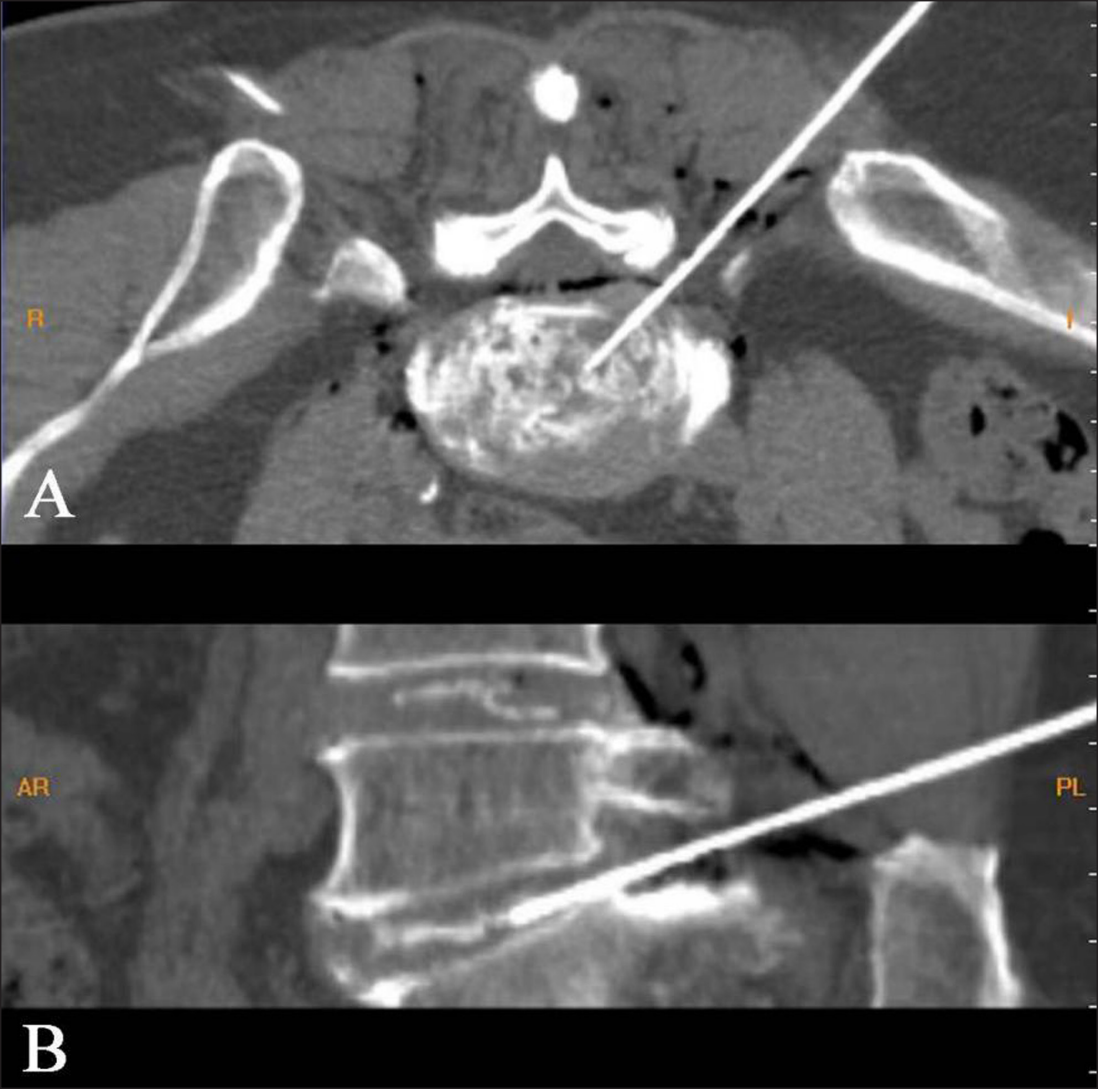
Intraoperative: Axial **(A)** and sagittal **(B)** CT images of collagenase treatment.

The patient was placed on the computed tomography (CT) table in a prone position, and then the doctor passed a needle along the lateral margin of the inferior articular process of the vertebra, and then through the neuroforamen into the disc. The position of the needle in the disc was confirmed on CT. The patients received 600 u collagenase via a needle (Figure 2). After surgery, the pain was relieved. Two days later, the patient was discharged.

### Recurrence and Resolution

**Figure 3:**
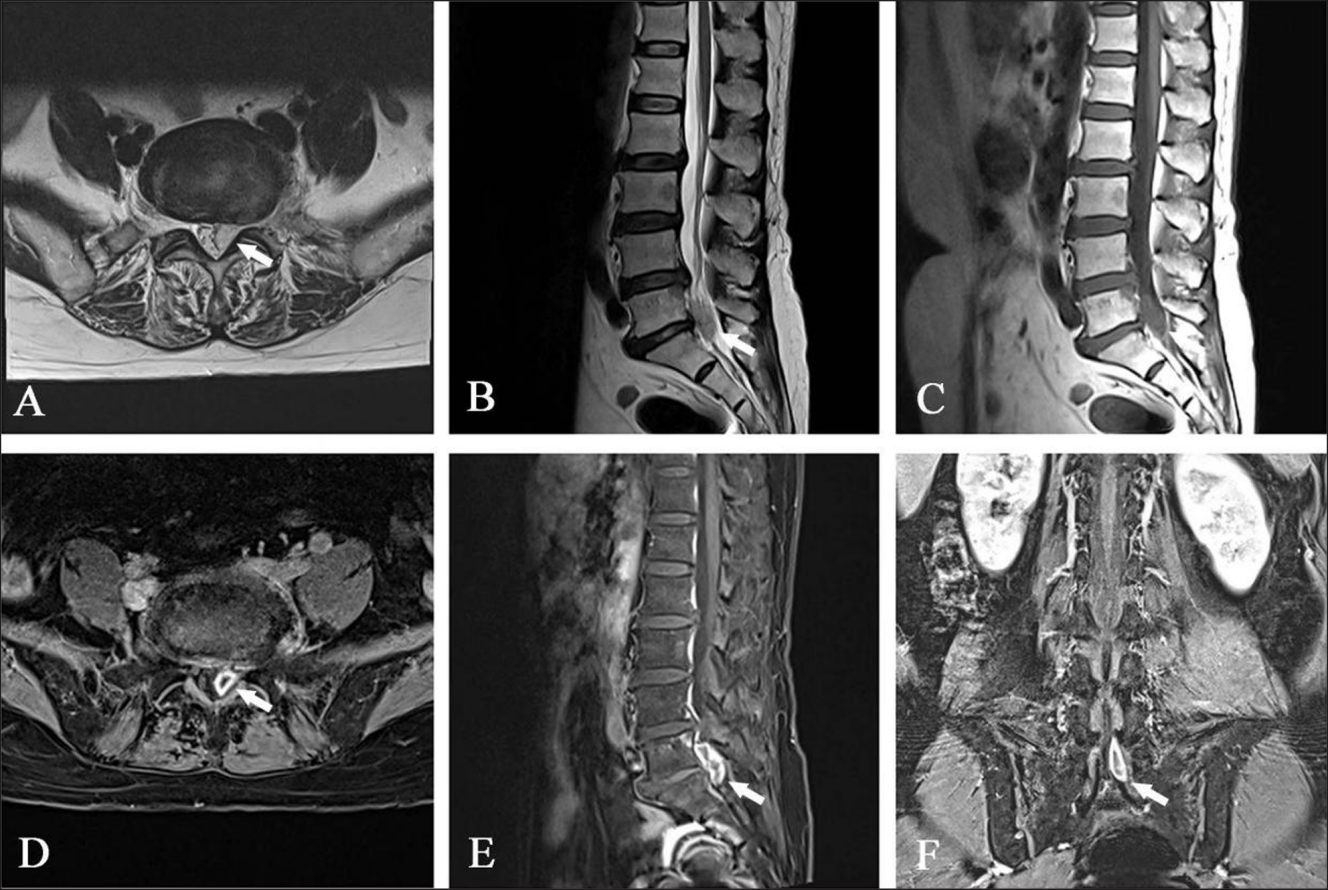
Postoperative: Axial T2-weighted MR image without contrast **(A)** shows a hyperintense nodule (arrow) in the spinal canal; sagittal image without contrast shows that the nodule (arrow) is hyperintense on T2WI **(B)** and isointense signal on T1WI **(C)**; fat-suppressed T1-weighted axial **(D)**, sagittal **(E)** and coronal **(F)** MR images show the nodule (arrow) displays a “ring” enhancement after gadolinium administration.

**Figure 4:**
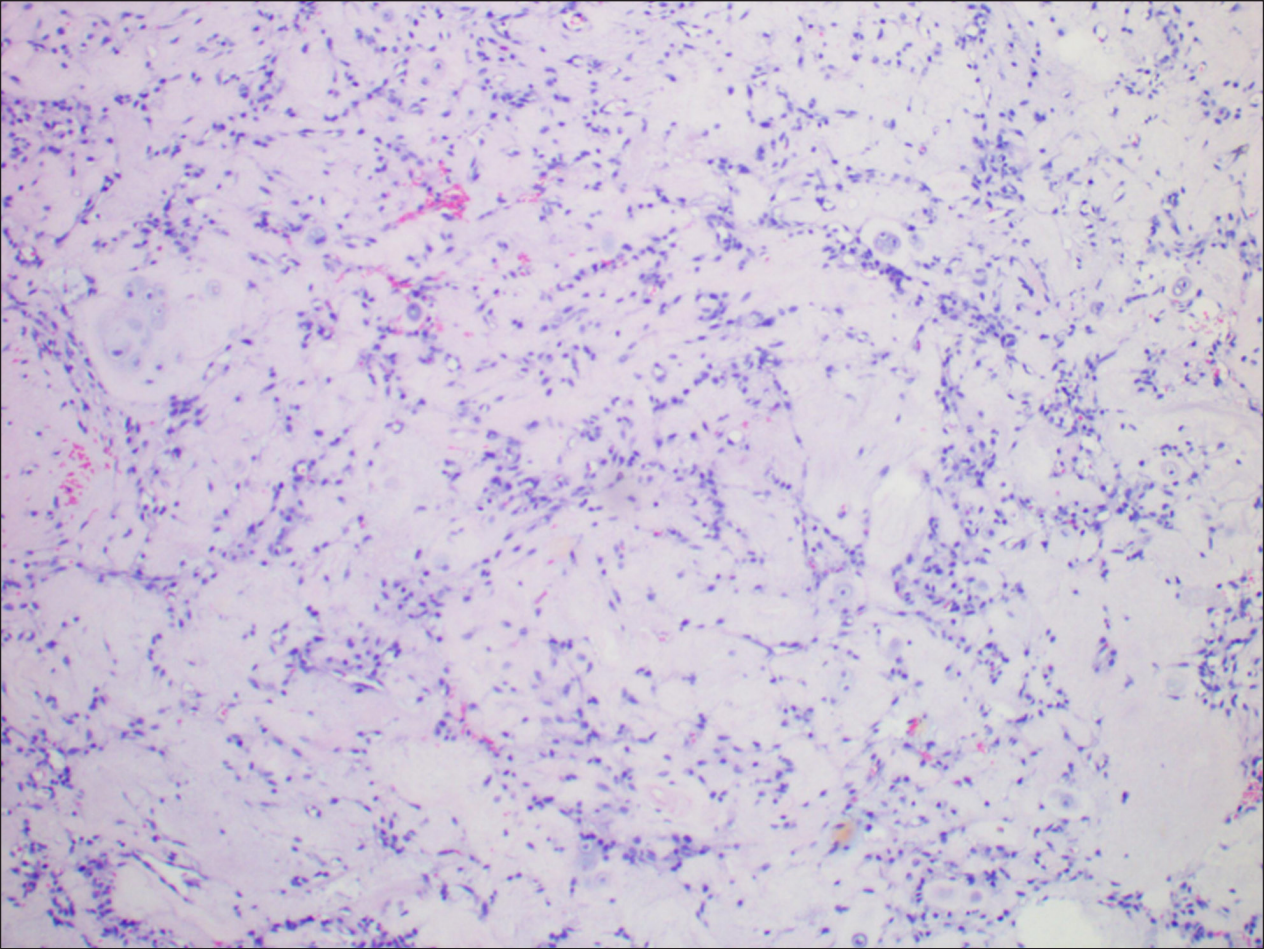
Pathology findings: The nudule was nucleus pulposus tissues, with some fibrous tissues and leukomonocytes.

Two weeks after discharge, the patient said she experienced pain in her lower left extremities after engaging in weight-bearing activity, this time accompanied by numbness. Her VAS pain score was 8. She had no fever, pain, or signs of meningeal irritation, and laboratory tests were normal. Lumbar MRI showed a nodule in the spinal canal at the L5–S1 level. It was 2.4 cm × 1.0 cm in size with isosignal on T1WI, hyperintense signal on T2WI (Figure 3 A-C), and a “ring” enhancement visible after administration of gadolinium (Figure 3 D-F). Two days later, the patient underwent surgical treatment (percutaneous endoscopic lumbar discectomy with an operative route of the posterolateral). Pathology showed that the nodule was nucleus pulposus tissue, with some fibrous tissues and leukomonocytes (Figure 4). Pain and numbness were significantly reduced after surgery.

## Discussion

The integrity failure of the annulus fibrosis can cause nucleus pulposus migrate into the spinal canal and cause anterior compression of the neural structures [5]. Collagenase can dissolve the nucleus pulposus tissues, especially collagen, at a certain pH and temperature. As the nucleus pulposus collagen fibers became damaged, swollen collagen fibers placed increased pressure on the disc, causing nucleus pulposus prolapsus [6].

The migrated nucleus pulposus without collagenase injection on the MRI image often appears an isosignal on T1WI, the hypointense signal on T2WI, and a thin “ring” enhancement after gadolinium administration. In our case, the migrated nucleus pulposus showed isosignal on T1WI and hyperintense signal on T2WI, and, after gadolinium-enhanced scanning, the rim of ring-shaped enhancement was relatively obvious. Histologic studies showed the proliferation of capillaries and numerous cellular elements around the migrated nucleus pulposus tissues.

## Conclusion

Many causes of nucleus pulposus herniation have been reported, the most common being degenerative changes. However, the migration of the nucleus pulposus after collagenase treatment has rarely been reported. Therefore, it should be included in the differential diagnosis in intradural–extramedullary tumors.
